# Influence of obturation technique and sealer type on the fracture resistance of maxillary central incisors with simulated traumatic fractures

**DOI:** 10.7717/peerj.21698

**Published:** 2026-09-14

**Authors:** Gözde Akbal Dinçer, Zeynep Buket Kaynar, Fatma Kaplan

**Affiliations:** 1 Department of Endodontics, Faculty of Dentistry, Istanbul Okan University, Istanbul, Turkey; 2 Department of Restorative Dentistry, Faculty of Dentistry, Istanbul Okan University, Istanbul, Turkey; 3 Department of Endodontics, Faculty of Dentistry, Istanbul Bezmialem University, Istanbul, Turkey

**Keywords:** Fracture resistance, Obturation technique, TotalFill BC, AH Plus, Calciumsilicate-based sealer, Traumatic dental injury

## Abstract

**Background:**

This study evaluated the effects of obturation technique and sealer type on the fracture resistance of endodontically treated maxillary central incisors with simulated traumatic crown fractures under clinically relevant loading conditions.

**Methods:**

Forty extracted human maxillary central incisors were randomly assigned to either the single-cone technique or warm vertical compaction technique. Each group was further divided according to the sealer used: AH Plus or TotalFill BC. After obturation, the crowns were restored with a short fiber-reinforced composite and a nanohybrid composite resin. The specimens were stored at 100% humidity for two weeks and embedded in acrylic resin. Fracture resistance was assessed using a universal testing machine with oblique loading at 45°. The data were analyzed using two-way analysis of variance (ANOVA) at a significance level of 0.05.

**Results:**

Both obturation technique and sealer type significantly affected fracture resistance. TotalFill BC produced higher fracture-load values than AH Plus, while the single-cone technique (SCT)/TotalFill BC group had the highest numerical mean. However, no significant interaction was found between obturation technique and sealer type.

**Conclusions:**

Within the limitations of this *in vitro* study, sealer selection emerged as the primary factor influencing fracture resistance. TotalFill BC provided greater fracture resistance than AH Plus across both obturation techniques, while the absence of a significant interaction indicated that neither technique conferred a sealer-specific advantage.

## Introduction

Endodontically treated teeth undergo substantial biomechanical changes, because of the loss of tooth structure during endodontic and restorative procedures and possible alterations in dentin properties  (Assif & Gorfil, 1994). These changes may reduce the ability of a tooth to withstand functional and traumatic loads, particularly in anterior teeth, in which crown morphology and the direction of force play important roles in stress distribution.

Severe fractures, including complicated crown or crown–root fractures represent a major clinical challenge after root canal treatment. Such fractures may compromise restorability and ultimately necessitate extraction. The fracture resistance of endodontically treated teeth is influenced by several factors, including the obturation technique and the physicochemical properties of the root canal sealer, both of which may affect the stress transmission and dissipation within root dentin  (Eren et al., 2021).

Epoxy resin-based sealers, such as AH Plus (Dentsply Maillefer, Tulsa, OK, USA), have long been considered a gold standard due to their favorable physicochemical properties, confirming their effectiveness as a sealing material. However, AH Plus lacks bioactive potential attributed to calcium silicate-based sealers (Rodríguez-Lozano et al., 2020). Calcium silicate-based sealers have been developed to enhance dentin–sealer interaction through bioactive mechanisms, including mineral deposition and chemical bonding at the sealer–dentin interface, which may influence the fracture resistance of treated teeth (Tay & Pashley, 2007).

TotalFill BC Sealer (FKG Dentaire, La Chaux-de-Fonds, Switzerland) is a premixed, hydrophilic calcium silicate-based material containing calcium silicate, monobasic calcium phosphate, calcium hydroxide, and zirconium oxide. It has been reported to exhibit lower cytotoxicity than AH Plus, together with greater biocompatibility, bioactivity, and lower porosity. In addition, it shows comparable penetration into lateral canals and is capable of forming hydroxyapatite, thereby promoting chemical bonding to dentin (Chan et al., 2024).

Obturation technique may also influence fracture resistance (Karapinar Kazandag et al., 2009). The single-cone technique (SCT) is widely used because of its simplicity and shorter chair time (Hargreaves, Berman & Rotstein, 2016); however, complete adaptation of gutta-percha to the canal walls may not always be achieved (Celikten et al., 2016). In contrast, the warm vertical compaction technique (WVCT) may improve adaptation and three-dimensional distribution of the filling material within the root canal system (Huang et al., 2018). Nevertheless, the heat and compaction forces applied during the WVCT may generate stresses within root dentin and potentially affect tooth strength (Hargreaves, Berman & Rotstein, 2016).

Maxillary central incisors are frequently affected by traumatic dental injuries and may present with extensive coronal tissue loss and an increased risk of subsequent fracture (Lembacher et al., 2022). Their rehabilitation requires restorative strategies that address both esthetic requirements and biomechanical reinforcement. EverX Posterior (GC Europe, Leuven, Belgium) is a short fiber-reinforced composite intended for use as a reinforcing substructure. By improving stress distribution and approximating the mechanical behavior of dentin, it has been proposed as a dentin-replacement material in regions exposed to substantial functional loads (Garoushi et al., 2018).

Most previous studies of fracture resistance have used decoronated specimens, an experimental design that may not adequately reproduce clinical force transmission (Dixit et al., 2025; Al-Hiyasat, Sawallha & Taha, 2023; Pandey et al., 2024; Lichaa et al., 2022; Ersoy & Evcil, 2015; Sagsen et al., 2007; Topçuoğlu et al., 2011). Evidence regarding the mechanical behavior of endodontically treated anterior teeth with residual coronal structure under clinically relevant oblique loading remains limited. Therefore, this study evaluated the effects of two obturation techniques (SCT and WVCT) and two root canal sealers (AH Plus and TotalFill BC) on the fracture resistance of endodontically treated maxillary central incisors with simulated traumatic crown fractures restored using a short fiber-reinforced composite resin (GC EverX Posterior) and nanohybrid composite resin (Estelite, Tokuyama Dental, Tokyo, Japan). The null hypothesis was that neither obturation technique nor sealer type would statistically affect fracture resistance.

## Materials & Methods

Ethical approval was obtained from the Scientific Research Ethics Committee of Bezmialem Vakıf University (Approval No: 2026/8, January 14, 2026). Written informed consent was obtained from all participants for the use of their extracted teeth for research purposes.

### Sample size calculation and tooth selection

The required sample size was calculated using G*Power software (version 3.1.9.6; Heinrich Heine University, Düsseldorf, Germany). With an alpha level of 0.05 and a statistical power of 80%, the calculation indicated that at least 10 specimens were required per group. Accordingly, each experimental group comprised 10 specimens (*n* = 10), and a total of 40 extracted human maxillary central incisors with comparable dimensions were included.

The extracted maxillary central incisors were selected from an institutional collection of teeth accumulated over time. The teeth had been extracted mainly for periodontal or prosthetic reasons. Following extraction, the teeth were disinfected and stored in 0.1% thymol solution at 4 °C until use. The storage period before experimentation did not exceed three months. To improve specimen standardization, only teeth with intact coronal and radicular structures and a total length of 22.0 ± 0.5 mm were selected. All specimens were examined under 25× magnification using a stereomicroscope (Zeiss, Heidelberg, Germany), and teeth with previous root canal treatment, caries, restorations, resorption, visible cracks, fractures, or structural defects were excluded.

### Specimen preparation

To simulate an Ellis Class III traumatic injury, the crowns were sectioned horizontally at the middle third using a diamond disk (IsoMet High Speed Pro; Buehler, Lake Bluff, IL, USA) under continuous water cooling (Pereira et al., 2023). Standard access cavities were prepared. The working length was determined using a size 10 K-file and established at one mm short of the apical foramen.

The root canals were prepared with ProTaper Next rotary instruments (Dentsply, Ballaigues, Switzerland) to size X4. During instrumentation, each canal was irrigated with two mL of 5% sodium hypochlorite after each instrument. The final irrigation protocol consisted of five mL 17% ethylenediaminetetraacetic acid followed by five mL of 5% sodium hypochlorite. The canals were then dried with paper points.

Specimens were randomly allocated to two main groups according to obturation technique: SCT and WVCT. Each main group was further divided according to the sealer used: AH Plus (Dentsply DeTrey, Konstanz, Germany) or TotalFill BC Sealer (FKG Dentaire, La Chaux-de-Fonds, Switzerland).

**SCT Group:** A size 40/.06 gutta-percha master cone (Dentsply Maillefer), coated with the assigned sealer, was inserted to the working length. Excess gutta-percha was removed and light vertical compaction was applied coronally.

**WVCT Group**: A size 40/.06 gutta-percha master cone (Dentsply Maillefer) coated with the assigned sealer, was inserted to the working length. The cone was down-packed to create a 3–4 mm apical filling using a Buchanan Heat Plugger Fine .06 connected to the Elements Free system (SybronEndo/Kerr Endodontics, Orange, CA, USA). Backfilling was performed with thermoplasticized gutta-percha injected at 200 °C in 4–5 mm increments and compacted using Buchanan hand pluggers (sizes 1 and 2).

After obturation, the crowns were restored with EverX Posterior as a short fiber-reinforced composite and nanohybrid composite resin overlay to reproduce the external morphology of a maxillary central incisor. The specimens were stored at 100% humidity for two weeks to allow the sealers to set completely. Each root was then embedded in acrylic resin, leaving two mm of the root surface exposed apical to the cementoenamel junction (CEJ).

### Fracture resistance test

**Figure 1:**
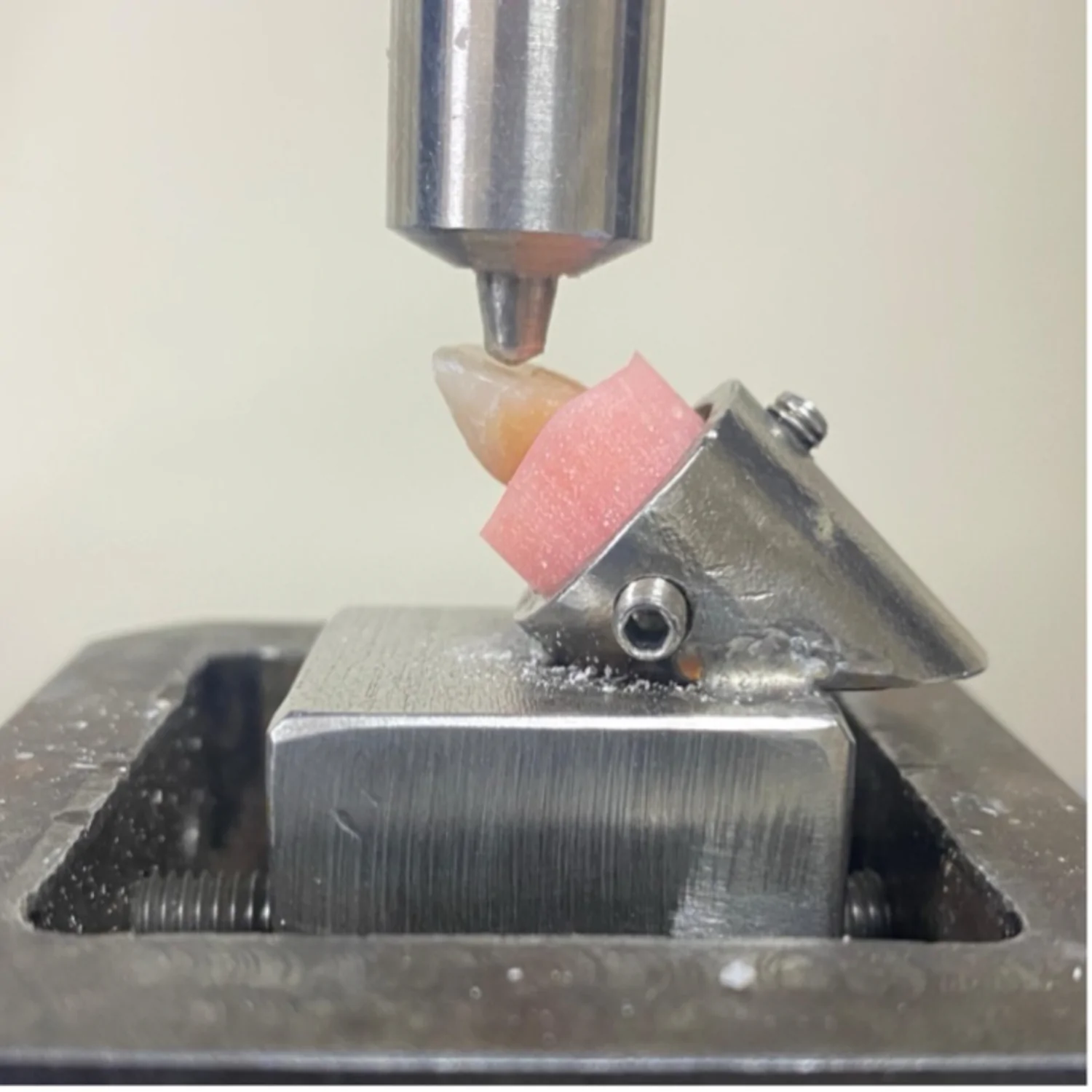
Experimental setup for fracture-resistance testing. Each specimen was embedded in acrylic resin and mounted on a custom-designed inclined metal platform. A compressive load was applied to the palatal surface at a 45° angle using a universal testing machine.

Each specimen was embedded in an acrylic block and mounted on a custom-designed inclined metal platform so that the palatal surface was positioned at an angle of 45° to the loading device (Fig. 1). A compressive load was applied to the palatal surface at a point two mm apical to the incisal edge using a universal testing machine (Instron Corp., MA, USA). Loading was performed with a four mm-diameter cylindrical tip at a crosshead speed of 1.0 mm/min. The maximum force required to induce fracture was recorded in Newtons (N).

Fracture testing was performed by an operator who was blinded to the group allocation. Specimens were identified using coded labels that did not disclose the obturation technique or sealer type. After fracture testing, fracture patterns were independently assessed by two calibrated examiners who were blinded to group allocation. Before the final assessment, the examiners reviewed the predefined criteria for repairable and irreparable fractures. Fractures confined to the crown or terminating at or coronal to the CEJ were classified as repairable. Fractures extending apical to the CEJ were classified as irreparable. Interexaminer agreement was evaluated using Cohen’s kappa coefficient and showed substantial agreement (*κ* = 0.744).

### Statistical analysis

Statistical analyses were performed using IBM SPSS Statistics, version 27 (IBM Corp., Armonk, NY, USA). Data normality was assessed using the Kolmogorov–Smirnov and Shapiro–Wilk tests, which indicated that the data followed a normal distribution. The effects of obturation technique and sealer type, as well as their potential interaction, on fracture resistance were evaluated using two-way analysis of variance (ANOVA). Differences among the four experimental groups were further evaluated using one-way ANOVA with Tukey’s HSD *post hoc* test. Differences in fracture-mode distribution among the groups were assessed using the Fisher–Freeman–Halton exact test. Effect sizes were reported as partial eta squared (*ηp*^2^) for two-way ANOVA and Hedges’ *g* for pairwise comparisons. Group means were also presented with 95% confidence intervals. Statistical significance was set at *p* < 0.05.

## Results

Two-way ANOVA showed statistically significant effect of obturation technique (*F* = 4.723, *p* = 0.036, *ηp*^2^ = 0.116) and sealer type (*F* = 77.627, *p* < 0.001, *ηp*^2^ = 0.683) on fracture resistance. However, the interaction between obturation technique and sealer type was not statistically significant (*F* = 0.581, *p* = 0.451, *ηp*^2^ = 0.016) (Table 1).

**Table 1:** Two-way ANOVA results evaluating the effects of obturation technique and sealer type on francture resistance.

**Source**	**Type III sum of squares**	**df**	**Mean square**	***F***	***p*-value**	*ηp* ^2^
Obturation Technique	84,517.04	1	84,517.04	4.723	0.036^*^	0.116
Sealer Type	1,389,147.00	1	1,389,147.00	77.627	<0.001^*^	0.683
Technique × Sealer	10,391.63	1	10,391.63	0.581	0.451	0.016

**Notes.**

* Two-way ANOVA test; *p* < 0.05 indicates statistical significance.

*ηp*^2^ partial eta squared df degrees of freedom

95% confidence intervals for group mean fracture-load values are presented in Table 2.

Within the SCT groups, specimens obturated with TotalFill BC Sealer showed significantly greater fracture resistance (809.81 ± 162.68 N) than those obturated with AH Plus (404.86 ±105.30 N; *p* = 0.001). Similarly, within the WVCT groups, specimens obturated with TotalFill BC (685.64 ± 166.34 N) demonstrated significantly greater fracture resistance than those obturated with AH Plus (345.16 ± 79.74 N; *p* = 0.001). Within the AH Plus groups, fracture resistance did not differ significantly between SCT and WVCT (*p* = 0.170). Similarly, within the TotalFill BC groups, no significant difference was detected between SCT and WVCT (*p* = 0.109) (Table 2).

**Table 2:** Evaluation of fracture resistance according to groups.

**Group**	**Fracture resistance (Mean ± SD, *N*)**	**95% CI**
Single-Cone–AH Plus	404.86 ± 105.30 ^A^	329.53–480.19
Single-Cone–TotalFill BC	809.81 ± 162.68 ^B^	693.43–926.18
Warm Vertical Compaction–AH Plus	345.16 ± 79.74 ^A^	288.12–402.20
Warm Vertical Compaction–TotalFill BC	685.64 ± 166.34 ^B^	566.64–804.63
*p*-value	0.001^*^	

**Notes.**

* One-way ANOVA test; *p* < 0.05 indicates statistical significance.

Values are presented as mean ± standard deviation and 95% confidence interval. Values sharing the same uppercase letter are not significantly different from each other. Values marked with different uppercase letters indicate statistically significant differences between the corresponding groups.

SCT single-cone technique WVCT warm vertical compaction technique CI confidence interval

A one-way ANOVA also demonstrated a significant difference in fracture resistance among the four experimental groups (*p* = 0.001). The SCT/TotalFill BC group had the highest mean fracture load and differed significantly from the SCT/AH Plus and WVCT/AH Plus groups (both *p* = 0.001). The WVCT/TotalFill BC group also showed significantly greater fracture resistance than AH Plus groups (both *p* = 0.001). No significant difference was detected between the SCT/AH Plus and WVCT/AH Plus groups (*p* = 0.170) or between the SCT/TotalFill BC and WVCT/TotalFill BC groups (*p* = 0.109) (Table 2). The pairwise effect sizes were large for the comparison between TotalFill BC and AH Plus within both the SCT groups (Hedges’ *g* = 2.83) and the WVCT groups (Hedges’ *g* = 2.50). The effect sizes for the comparisons between SCT and WVCT were moderate within the AH Plus groups (Hedges’ *g* = 0.61) and the TotalFill BC groups (Hedges’ *g* = 0.72).

**Figure 2:**
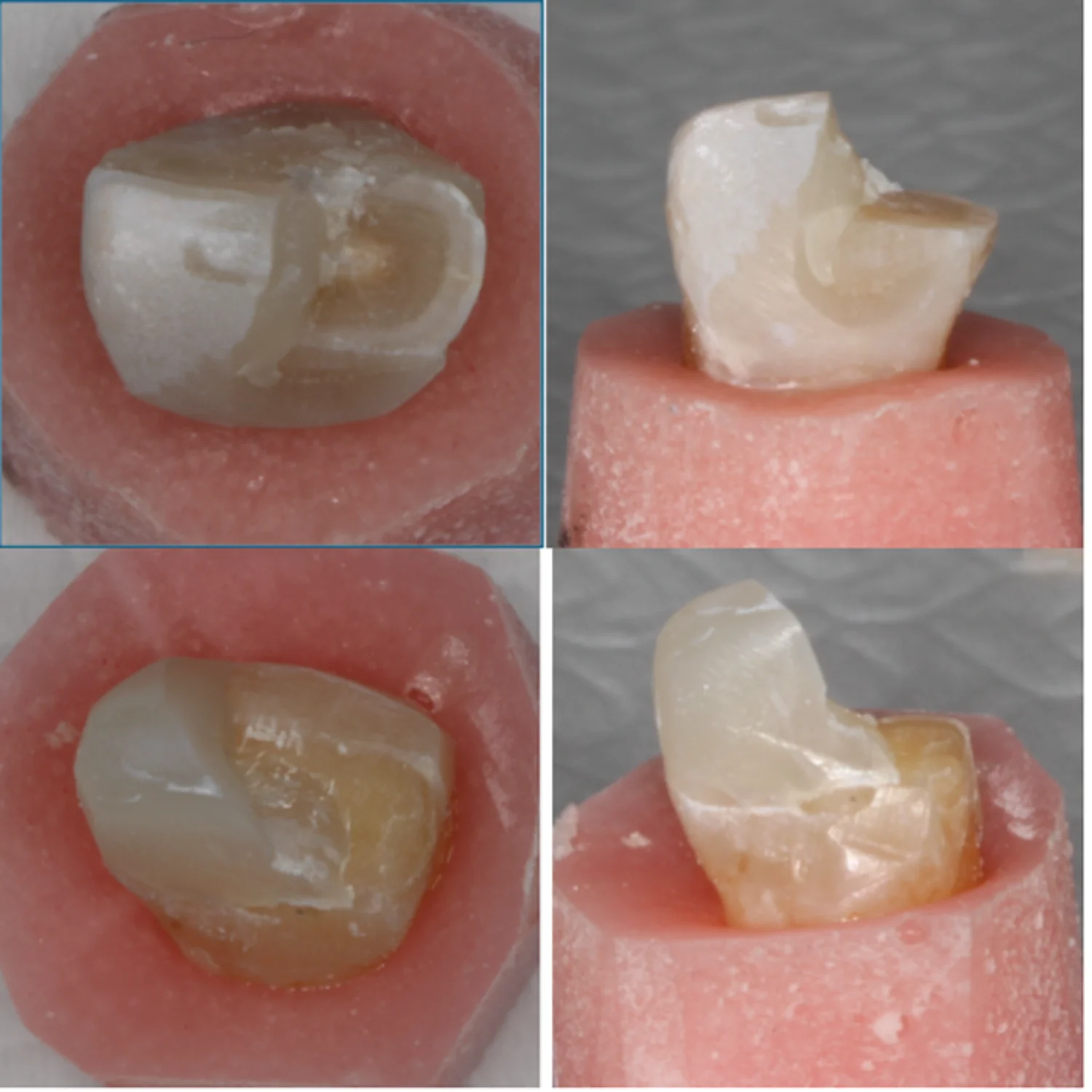
Representative repairable fracture patterns observed after fracture-resistance testing in Single Cone-AH Plus Group. In all specimens shown, the fracture lines were confined to the crown or terminated at or coronal to the cementoenamel junction (CEJ); therefore, the fractures were classified as repairable.

**Figure 3:**
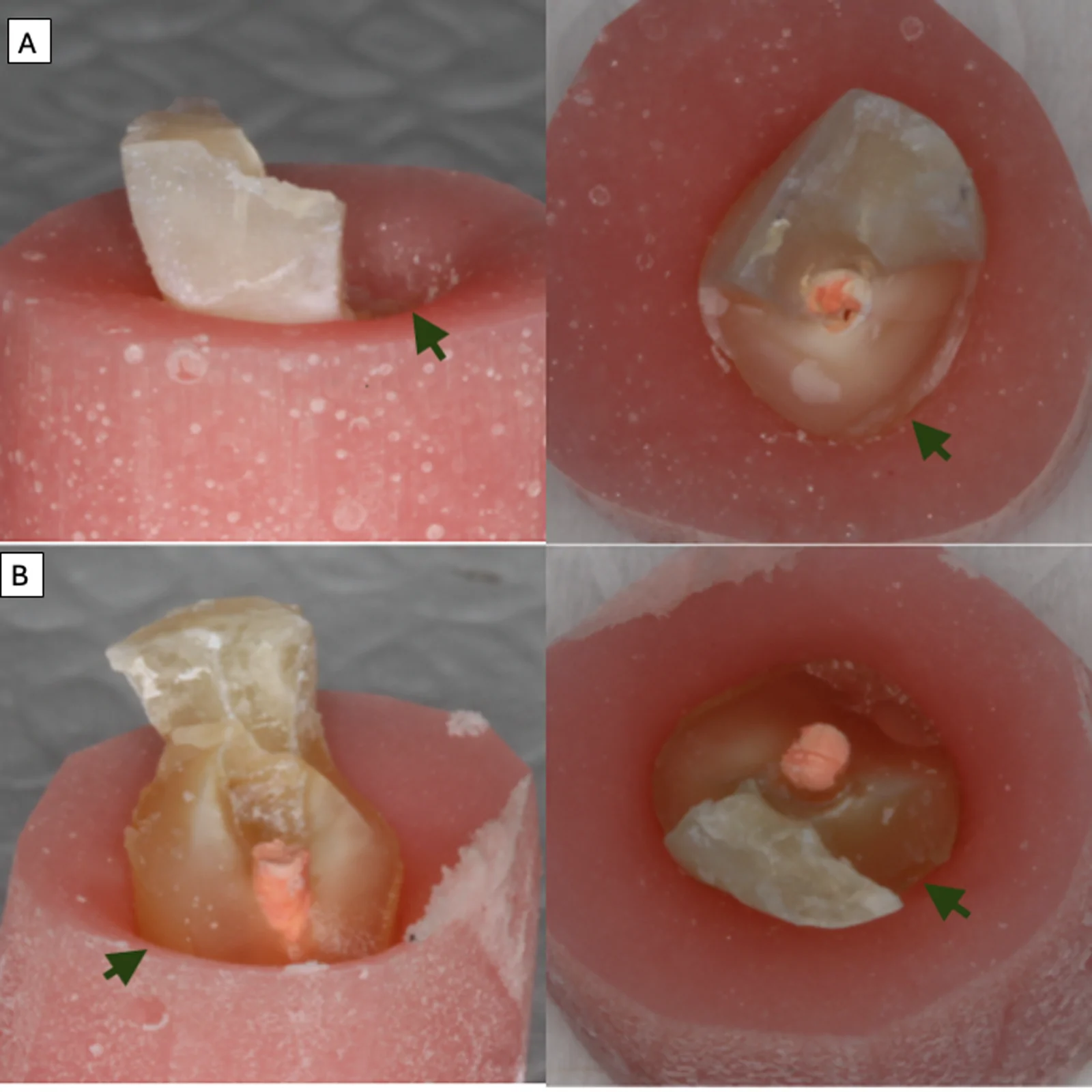
Representative irreparable fracture patterns observed after fracture-resistance testing. (A) Irreparable fractures in the warm vertical compaction technique (WVCT)/AH Plus group. (B) Irreparable fractures in the WVCT/TotalFill BC group. In all specimens shown, the fracture lines extended apical to the cementoenamel junction (CEJ), and the fractures were therefore classified as irreparable. Arrows indicate the fracture lines.

Fracture mode distribution did not differ significantly among the groups (*p* = 0.477). Repairable fractures occurred in 50% of specimens in both the SCT/AH Plus and SCT/TotalFill BC groups, 20% of specimens in the WVCT/AH Plus group, and 30% of specimens in the WVCT/TotalFill BC group (Table 3, Figs. 2 and 3).

**Table 3:** Distribution of fracture types among groups.

**Group**	**Repairable *n* (%)**	**Irreparable *n* (%)**	***p*-value**
Single Cone–AH Plus	5 (50%)	5 (50%)	
Single Cone–TotalFill BC	5 (50%)	5 (50%)	
Warm Vertical Compaction–AH Plus	2 (20%)	8 (80%)	
Warm Vertical Compaction–TotalFill BC	3 (30%)	7 (70%)	0.477^*^

**Notes.**

* Fisher–Freeman–Halton Exact Test; *p* > 0.05 indicates no significant difference.

## Discussion

This study evaluated the effects of obturation technique and sealer type on the fracture resistance of endodontically treated maxillary central incisors with standardized traumatic crown fractures. Both factors had significant main effects, whereas their interaction was not significant; therefore, the null hypothesis was partially rejected. The effect-size analysis further indicated that sealer type had a greater influence on fracture resistance than obturation technique. The large partial eta squared value for sealer type and the large pairwise effect sizes observed between TotalFill BC and AH Plus within both obturation techniques support the biomechanical relevance of this finding. Accordingly, TotalFill BC was associated with greater fracture resistance than AH Plus in both obturation groups. The SCT/TotalFill BC group showed the highest numerical mean fracture load; however, given the absence of a significant technique-by-sealer interaction and the very small interaction effect size, this finding should be interpreted as a numerical observation rather than evidence of a synergistic or combination-specific effect. Although the sample size was determined by an *a priori* power analysis, the use of 10 specimens per group and the inherent biological variability of extracted human teeth may have reduced the sensitivity to detect small or moderate interaction effects.

Maxillary central incisors are frequently affected by traumatic dental injuries because of their anterior position and may develop complicated crown or crown–root fractures after direct impact (Lembacher et al., 2022; Ramachandran et al., 2021). These teeth are important for esthetics and phonetics, and preserving their structural integrity is a major clinical objective. In addition, their relatively uniform single-rooted anatomy provides a suitable model for standardized evaluation of obturation techniques and sealer types. For these reasons, maxillary central incisors were selected for the present study.

The crowns were sectioned at the mid-coronal level to simulate an Ellis Class III fracture, which is characterized by a complicated crown fracture with pulp exposure (Pereira et al., 2023). This standardized approach reproduced substantial coronal tissue loss while preserving sufficient residual structure for restoration and load application. Although *in vivo* traumatic fractures may present with irregular and unpredictable fracture lines, irregular margins are often regularized and bevelled during restorative management to improve bonding and esthetic transition. Therefore, this model may represent a clinically relevant restorative procedure; however, it should not be interpreted as an exact reproduction of the initial traumatic fracture pattern.

EverX Posterior is a short-fiber-reinforced composite designed to approximate the mechanical behavior of dentin. The biomimetic restorative approach of using a short fiber-reinforced composite as a substructure beneath a conventional composite overlay has been recommended for teeth exposed to high functional stress (Garoushi et al., 2018). In the present study, this material was used as a reinforcing substructure to improve stress distribution within the restored crowns.

In several previous studies, the teeth were decoronated and loads were applied perpendicular to the long axis of the root (Dixit et al., 2025; Al-Hiyasat, Sawallha & Taha, 2023; Pandey et al., 2024; Lichaa et al., 2022; Ersoy & Evcil, 2015; Sagsen et al., 2007; Topçuoğlu et al., 2011), partly because oblique loading may produce nonuniform stress distribution and increase experimental variability (Lichaa et al., 2022). However, a finite element analysis comparing vertical and 45° oblique loading found no significant difference in stress distribution in mandibular teeth  (Yuan et al., 2016). Decoronation may alter natural force transmission and limit the clinical relevance of a fracture-resistance model. Therefore, in the present study, the standardized residual coronal structure was restored and retained during testing, and the load was applied to the palatal surface at an angle of 45° to approximate the direction of forces acting on maxillary anterior teeth.

The fracture-load values obtained in the present study should also be interpreted in relation to the functional forces acting on anterior teeth. The mean fracture-load values ranged from approximately 345 to 810 N. Reported measured maximal incisal forces in adults are approximately 325–382 N (Waltimo & Könönen, 1995), whereas model-based estimates of maximum possible incisal bite force have ranged from approximately 687 to 743 N  (Mainland & Hiltz, 1934; Osborn & Baragar, 1985; Koolstra et al., 1988). In this context, the fracture-load values in the AH Plus groups were close to the measured maximal incisal-force range, whereas those in the TotalFill BC groups approached or exceeded the higher estimated incisal-force range. However, the present values represent single-load failure thresholds under static *in vitro* conditions. Clinically, repeated cyclic forces of lower magnitude may induce fatigue-related crack propagation and lead to fracture over time. Therefore, these findings may be biomechanically meaningful, but their long-term clinical significance should be confirmed by studies incorporating cyclic loading, thermocycling, and aging protocols.

TotalFill BC sealer was selected because of its slight expansion during setting, compatibility with the SCT (Gandolfi et al., 2009), and reported thermal stability, which permits its use with warm obturation techniques (Qu et al., 2016). Several studies have reported greater fracture resistance with calcium silicate-based sealers than with epoxy resin-based sealers such as AH Plus (Mohammed & Al-Zaka, 2013; Kashaf et al., 2024; Osiri et al., 2018). Song et al. (2025) reported the highest fracture resistance when a bioceramic sealer was used with the single-cone technique. This effect has been attributed to hydroxyapatite formation, potential chemical interaction with dentin, penetration into dentinal tubules and improved adaptation to the root canal wall. In one study, scanning electron microscopy demonstrated close adaptation and dentinal tubule penetration of TotalFill BC, whereas interfacial gaps with no penetration were observed with AH Plus (Al-Hiyasat, Sawallha & Taha, 2023). Moreover, Osiri et al. (2018) reported fracture-strength values comparable to those of intact teeth when TotalFill BC was used with bioceramic-coated gutta-percha cones. Although such cones were not used in the present study, TotalFill BC was associated with higher fracture-load values than AH Plus in both obturation groups.

In contrast to our findings, Alkahtany et al. (2021) reported comparable vertical root fracture resistance for roots obturated with TotalFill BC and AH Plus, regardless of the obturation technique. Salim & Saleem (2025) reported greater fracture resistance with AH Plus and AH Plus BC than with TotalFill BC and higher values with thermoplasticized obturation techniques than with the single-cone technique. These conflicting findings may be explained by differences in tooth type, specimen decoronation and loading direction. A recent systematic review and network meta-analysis of *in vitro* studies, including 48 trials and 2,724 single-canal roots, reported favorable rankings for calcium silicate-based obturation systems and identified the SCT as the obturation technique with the highest vertical root fracture resistance  (Li et al., 2024). Nevertheless, the authors emphasized the substantial heterogeneity of *in vitro* study designs and the need for caution when extrapolating these findings to clinical conditions.

Previous studies have reported higher fracture-resistance values for SCT than for WVCT (Dixit et al., 2025; Al-Hiyasat, Sawallha & Taha, 2023; Pandey et al., 2024). The SCT may introduce less thermal and compaction-related stress into root dentin. In contrast, the vertical pressure used during WVCT (Shemesh et al., 2009) and the associated heat may affect both dentin and sealer properties. Heat has been reported to reduce the setting time and compressive strength of AH Plus, possibly by accelerating polymerization through changes in its amine components, whereas the chemical structure of calcium silicate-based sealers appears to be less affected (Atmeh & AlShwaimi, 2017). These differences may partly explain why TotalFill BC produced higher fracture-load values than AH Plus within the WVCT groups.

Irreparable fractures occurred numerically more often in the WVCT groups than in the SCT groups. The combination of vertical compaction pressure and heat application in the WVCT may generate additional stress within the root dentin, promoting microcrack formation and reducing structural integrity. However, fracture-mode distribution did not differ significantly among the four groups. Accordingly, this numerical pattern should be interpreted cautiously and should not be regarded as evidence that WVCT independently increases the risk of unfavorable fracture.

This study has several limitations. Fracture resistance was assessed using a single continuously increasing static load; therefore, the model did not reproduce the repetitive, multidirectional, and time-dependent forces or thermal aging that occur clinically. The absence of thermocycling, cyclic fatigue loading, periodontal ligament simulation, and intact or instrumented-but-unobturated control groups limits the direct clinical extrapolation of the findings. These limitations should be considered when interpreting the findings.

## Conclusions

Within the limitations of this *in vitro* study, sealer type appeared to exert a greater influence on fracture resistance than obturation technique, with TotalFill BC yielding higher fracture loads than AH Plus. Although the SCT/TotalFill BC group exhibited the highest numerical mean, the lack of a significant technique-by-sealer interaction does not support a synergistic effect. Fracture-mode distributions did not differ significantly among the experimental groups; therefore, the observed numerical differences in repairable and irreparable fractures should be interpreted cautiously.

##  Supplemental Information

10.7717/peerj.21698/supp-1 Supplemental Information 1 Similarty report

10.7717/peerj.21698/supp-2 Supplemental Information 2 Experimental results

10.7717/peerj.21698/supp-3 Supplemental Information 3 Distribution of fracture types (%) among the groups

## Additional Information and Declarations

### Data Availability

The following information was supplied regarding data availability: The raw measurements are available in the Supplemental Files.
