# Supplementary material for: Influence of obturation technique and sealer type on the fracture resistance of maxillary central incisors with simulated traumatic fractures
Source: PeerJ. 2026 Sep 14;14:e21698. doi: 10.7717/peerj.21698 (PMC13584932; doi:10.7717/peerj.21698)
Supplement: Supplementary file 1 [file peerj-14-21698-s001.pdf]

dolum tekniği̇ni ve kullanılan patın kırılmaya etkisi turnitin.docx

İstanbul Okan Üniversitesi

Document Details

Submission ID

trn:oid:::3117:545431402

Submission Date

Jan 12, 2026, 2:06 PM GMT+3

Download Date

Jan 12, 2026, 2:08 PM GMT+3

File Name

dolum tekniği̇ni ve kullanılan patın kırılmaya etkisi turnitin.docx

File Size

28.5 KB

8 Pages

2,510 Words

14,762 Characters

21% Overall Similarity

The combined total of all matches, including overlapping sources, for each database.

Filtered from the Report

- Bibliography
- Small Matches (less than 8 words)
- Abstract

Match Groups

- **42** Not Cited or Quoted 19%
  Matches with neither in-text citation nor quotation marks
- **4** Missing Quotations 2%
  Matches that are still very similar to source material
- **0** Missing Citation 0%
  Matches that have quotation marks, but no in-text citation
- **0** Cited and Quoted 0%
  Matches with in-text citation present, but no quotation marks

Top Sources

16% Internet sources
16% Publications
0% Submitted works (Student Papers)

Integrity Flags

0 Integrity Flags for Review

No suspicious text manipulations found.

Our system's algorithms look deeply at a document for any inconsistencies that would set it apart from a normal submission. If we notice something strange, we flag it for you to review.

A Flag is not necessarily an indicator of a problem. However, we'd recommend you focus your attention there for further review.

Match Groups

- **42** Not Cited or Quoted 19%
  Matches with neither in-text citation nor quotation marks
- **4** Missing Quotations 2%
  Matches that are still very similar to source material
- **0** Missing Citation 0%
  Matches that have quotation marks, but no in-text citation
- **0** Cited and Quoted 0%
  Matches with in-text citation present, but no quotation marks

Top Sources

16% Internet sources
16% Publications
0% Submitted works (Student Papers)

Top Sources

The sources with the highest number of matches within the submission. Overlapping sources will not be displayed.

#	Type	Source	%
1	Internet	www.bioinformation.net	2%
2	Internet	www.mdpi.com	1%
3	Internet	www.researchsquare.com	1%
4	Publication	Barış ÇELİK, Ayla YAVA, Aynur KOYUNCU, Zerrin ÇİĞDEM. "Investigation of the Rel...	1%
5	Publication	Tuba Gok, Ismail Davut Capar, Ilgin Akcay, Ali Keles. "Evaluation of Different Tech...	<1%
6	Internet	5dok.net	<1%
7	Internet	qa1.scielo.br	<1%
8	Publication	Michael Naumann, Anja Preuss, Martin Rosentritt. " Effect of incomplete crown fe...	<1%
9	Internet	journals.lww.com	<1%
10	Publication	Mehran Falahchai, Hamid Neshandar Asli, Mehdi Daneshvar, Amirreza Hendi. "Eff...	<1%

11 Publication

Vasfiye Isik, Soner Sismanoglu. "Managing Failed Fragment Reattachment: Influe... <1%

12 Publication

İkbal Sena Çelebi Keskin, Hilal Kabadayı, H. Seda Vatansever, Fehmi Raif Erişen. "I... <1%

13 Publication

Kang, Eunsoo. "Exploring Visual Word Recognition in Late Second Language Lear... <1%

14 Internet

bmcpalliatcare.biomedcentral.com <1%

15 Publication

H.R. Bakhsheshi-Rad, E. Hamzah, Mark P. Staiger, George J. Dias, Z. Hadisi, M. Sah... <1%

16 Publication

K. Gulsahi. "Sealer area associated with cold lateral condensation of gutta-percha... <1%

17 Internet

uca.hal.science <1%

18 Publication

Kali Vo, Joan Daniel, Chul Ahn, Carolyn Primus, Takashi Komabayashi. "Coronal an... <1%

19 Internet

www.jemds.com <1%

20 Publication

Sittichoke Osiri, Danuchit Banomyong, Vanthana Sattabanasuk, Kallaya Yanpiset.... <1%

21 Publication

Zandbiglari, T.. "Influence of instrument taper on the resistance to fracture of e... <1%

22 Internet

journal.uod.ac <1%

23 Internet

link.springer.com <1%

24 Internet

pure-oai.bham.ac.uk <1%

25 Publication

LG Belizario, TL Piragine, AC Girotto, MB Gelio, JR Pereira, E Fernandez, MC Kuga. ... <1%

26 Publication

M. Venturi. "A preliminary analysis of the morphology of lateral canals after root ... <1%

27 Publication

Ruiqi Yang, Jun Tian, Xiangya Huang, Shuxiang Lei, Yanling Cai, Zhezhen Xu, Xi W... <1%

28 Internet

dbc.wroc.pl <1%

29 Internet

pmc.ncbi.nlm.nih.gov <1%

30 Internet

www.nature.com <1%

31 Publication

F. Monticelli. "Sealing properties of two contemporary single-cone obturation sys... <1%

32 Publication

Kakamari, Sapna. "A Comparative Evaluation of the Effect of 8% and 17% Ethylen... <1%

33 Publication

Soner Sismanoglu, Vasfiye Isik. "Reattachment or Restoration? Fracture Resistanc... <1%

34 Internet

www.grafiati.com <1%

35 Internet

www.ijoprd.com <1%

36 Internet

www.ncbi.nlm.nih.gov <1%

8 **Influence of Obturation Technique and Sealer Type on the Fracture Resistance of Maxillary Central Incisors with Simulated Traumatic Fractures (Effect of Obturation Technique and Sealer on Fracture Resistance)**

Introduction

Endodontic treatment leads to substantial changes in the biomechanical behavior of teeth as a result of reduced dentin moisture (2) and the loss of tooth structure during endodontic and restorative procedures (3). These alterations may reduce the ability of the tooth to withstand functional and traumatic loads, particularly in anterior teeth, where force direction and crown morphology play a critical role in stress distribution.

Among the complications observed following root canal treatment, severe fracture patterns such as complicated crown or crown–root fractures represent a significant clinical challenge. These fractures often compromise restorability and may ultimately necessitate tooth extraction (4). The resistance of endodontically treated teeth to fracture is influenced by several factors, including the obturation approach and the physicochemical characteristics of the root canal sealer, both of which affect the transmission and dissipation of stresses within root dentin (5).

24 The epoxy resin–based sealers, exemplified by AH Plus (Dentsply Maillefer, Tulsa, OK, USA) have long been considered a gold standard due to their excellent physicochemical properties, confirming its effectiveness as a sealing material (6), although it lacks bioactive potential (7). In contrast, calcium silicate–based sealers have been introduced with the aim of enhancing dentin–sealer interaction through bioactive mechanisms, such as mineral deposition and chemical bonding at the sealer–dentin interface, which may improve the overall fracture resistance of treated teeth (8).

Recently, TotalFill BC (FKG Dentaire, Switzerland), has been introduced. This hydrophilic
19 material contains calcium silicate, calcium phosphate monobasic, calcium hydroxide, and zirconium oxide (9). It has been shown to exhibit lower cytotoxicity than AH Plus, with greater biocompatibility, bioactivity, and reduced porosity (10). In addition, it demonstrates
28 comparable penetration into lateral canals and the ability to form hydroxyapatite, enabling a chemical bond with dentin (11).

The obturation technique is another factor influencing fracture resistance (12). The single-cone technique (SCT) is widely used because of its simplicity and reduced chair time (13); however, complete adaptation of the gutta-percha along the canal walls may not always be achieved (14). In contrast, the warm vertical compaction technique (WVCT) allows improved adaptation of the filling material, producing a more homogeneous and three-dimensional seal (15). Despite these advantages, the heat and compaction forces applied during the WVCT technique may generate internal stresses within the root dentin, potentially compromising tooth strength (13).

Maxillary central incisors are the teeth most frequently affected by trauma, often presenting with extensive coronal tissue loss and a higher risk of fracture (16). Successful rehabilitation of these teeth requires restorative strategies that address not only esthetic demands but also biomechanical reinforcement. EverX Posterior (GC Europe) is a fiber-reinforced composite formulated with polyethylene and glass fibers for reinforcement of endodontically treated teeth. By enhancing stress distribution and mimicking the biomechanical response of dentin, it has been proposed as a bulk base material in regions subjected to high masticatory forces (17).

Most previous investigations evaluating fracture resistance have relied on decoronated specimens, a design that may not adequately reflect clinical conditions (18-24). Information regarding the behavior of endodontically treated anterior teeth with preserved crown structure under intraoral-like loading remains limited. In this context, the present study was designed to analyze the influence of two different obturation techniques (SCT and WVCT) and two root canal sealers (AH Plus and TotalFill BC) on the fracture resistance of endodontically treated maxillary central incisors, with simulated traumatic fractures, restored using a fiber-reinforced composite resin (GC EverX Posterior) combined with nano-hybrid composite resin (Tokuyama Estelite, Tokuyama Dental, Tokyo, Japan). The null hypothesis was that variations in obturation technique or sealer type would not result in statistically significant differences in fracture resistance.

Materials and Methods

The study protocol was reviewed and approved by the Bezmialem University Research Ethics Committee (number).

Sample Size Calculation and Tooth Selection

4 The required sample size was calculated using G*Power software (version 3.1.9.6; Heinrich Heine University, Düsseldorf, Germany), assuming an alpha error of 0.05 and a statistical power of 80%, which indicated that at least 10 specimens were needed for each group. A total of forty extracted human maxillary central incisors with comparable dimensions were included.

25 Following extraction, the specimens were disinfected and preserved in a 0.1% thymol solution at 4°C until further use. To ensure uniformity among specimens, teeth with a length of 22.0 ± 0.5 mm were selected. All specimens were further inspected under 25× magnification using a stereomicroscope (Zeiss, Heidelberg, Germany) to exclude teeth with cracks, fractures or structural defects.

Specimen Preparation

To simulate traumatic injury (Ellis class III fracture), the crowns were sectioned horizontally
11 at the middle third using a diamond disk (IsoMet High Speed Pro; Buehler, Lake Bluff, IL, USA) under continuous water cooling. (25,26) Standart access cavities were prepared and working length determination was performed by introducing a #10 K-file into the canal and
7 setting the length at 1 mm short of the apical foramen.

22 Root canal preparation was performed with ProTaper Next Rotary files (Dentsply, Ballaigues,
3 Switzerland) up to size X3. During preparation, canals were irrigated with 2 ml of %2.5
18 NaOCl following each file. Final irrigation consisted of 5 mL 17% EDTA followed by 5 mL of 5% NaOCl, after which canals were dried with paper points.

30 Specimens were randomly allocated into two main groups according to obturation technique:
9 single-cone technique and warm vertical compaction technique. Each group was further
12 divided based on the sealer used: AH Plus (Dentsply DeTrey, Konstanz, Germany) or TotalFill BC Sealer (FKG Dentaire, La Chaux-de-Fonds, Switzerland).

16 SCT Group: A size 30/.06 taper gutta-percha master cone (Dentsply Maillefer) coated with sealer was placed to the working length. Excess gutta-percha was removed and light vertical compaction was applied coronally.

5 WVCT Group: A size 30/.06 taper gutta-percha master cone (Dentsply Maillefer) was
5 positioned similarly and down-packed to create a 3–4 mm apical fill using Buchanan Heat Plugger Fine .06 (SybronEndo/Kerr Endodontics, Orange, CA) connected to the Elements

Free (SybronEndo/Kerr Endodontics, Orange, CA). Backfill was completed with thermoplasticized gutta-percha injected at 200°C using the Elements system in 4–5 mm increments and compacted using Buchanan Hand Pluggers (sizes 1 and 2).

After obturation, the coronal restoration was completed using EverX Posterior and nano-hybrid composite to mimic the morphology of an intact maxillary central incisor. Specimens were stored at 100% humidity for two weeks to allow complete sealer setting. Each root was then embedded in acrylic resin, leaving 2 mm of the root surface exposed below the cementoenamel junction.

Fracture Resistance Test

Each specimen was embedded in an acrylic block and mounted on a custom-designed inclined metal platform, allowing the palatal surface of the tooth to be oriented at an angle of 45° relative to the loading device (Figure 1). Mechanical loading was applied to the palatal surface at a point located 2 mm apical to the incisal edge using a universal testing machine (Instron Corp., MA, USA). The load was delivered at a constant crosshead speed of 1.0 mm/min through a cylindrical tip with a diameter of 4 mm. The maximum force required to induce fracture was recorded for each specimen and expressed in Newtons (N).

Statistical Analysis

Statistical evaluation was performed using IBM SPSS Statistics software (version 27; IBM Corp., Armonk, NY, USA). Data distribution was assessed for normality using the Kolmogorov–Smirnov and Shapiro–Wilk tests, which indicated that the data followed a normal distribution. The effects of obturation technique and sealer type, as well as their potential interaction, on fracture resistance were analyzed using two-way analysis of variance (ANOVA). Differences in fracture pattern distribution among the groups were examined with the Fisher–Freeman–Halton exact test. Statistical significance was defined as a p-value less than 0.05.

Results

Two-way ANOVA revealed a statistically significant effect of obturation technique and sealer type on fracture resistance ($p = 0.036$) and sealer type ($p = 0.001$). However, the interaction between obturation technique and sealer type was not statistically significant ($p = 0.451$). (Table 1)

When the SCT was used, specimens obturated with TotalFill BC Sealer showed significantly higher fracture resistance (809.81 ± 162.68 N) than those obturated with AH Plus (404.86 ± 105.30 N) ($p = 0.001$). Similarly, in the WVCT groups, specimens filled with TotalFill BC Sealer (685.64 ± 166.34 N) demonstrated significantly greater fracture resistance compared with those obturated with AH Plus (345.16 ± 79.74 N) ($p = 0.001$). Within each sealer type,
35 no significant difference in fracture resistance was found between obturation techniques ($p > 0.05$). (Table 2)

10 One-way ANOVA indicated a significant difference in fracture resistance among the four groups ($p = 0.001$). The SCT/TotalFill BC group recorded the highest mean fracture load,
23 significantly higher than both the SCT/AH Plus and WVCT/AH Plus groups ($p < 0.05$). The WVCT/TotalFill BC group also exhibited significantly greater fracture resistance compared
17 with the AH Plus groups ($p < 0.05$). No significant difference was observed between groups using the same sealer ($p > 0.05$). (Table 2)

2 No significant difference was found in fracture mode distribution among groups ($p > 0.05$). Repairable fractures were observed in 50% of specimens in both the SCT/AH Plus and
26 SCT/TotalFill BC groups, 20% in the WVCT/AH Plus group, and 30% in the WVCT/TotalFill BC group. (Table 3, Figure 2, Figure 3)

Discussion

Maxillary central incisors are the teeth most frequently affected by traumatic injuries (16). Their anterior position and single-rooted anatomy make them highly susceptible to direct impact during accidents or falls, often leading to complicated crown or crown–root fractures (27). These teeth are essential for esthetics and phonetics, and preserving their structural integrity is a primary clinical concern (28). Moreover, their uniform canal morphology also provides a standardized model for evaluating obturation techniques and sealer types. For these
33 reasons, maxillary central incisors were selected in the present study.

The crowns were sectioned at the mid-coronal level to simulate an Ellis Class III fracture, representing a complicated crown fracture with pulp exposure (26). This approach provided a standardized and clinically relevant model that reproduced the structural loss seen in traumatized anterior teeth while maintaining sufficient coronal structure for restoration and load application.

EverX Posterior, a short-fiber–reinforced composite designed to mimic the mechanical properties of dentin. Because the biomimetic restorative technique—using short fiber–reinforced composite as a substructure with a conventional composite overlay—has been recommended for restoring teeth in high stress-bearing areas (17), this material was used in the present study to strenght the teeth, simulating their pre-trauma condition. This restorative technique improves stress absorption and load-bearing capacity, thereby contributing to the overall reinforcement of endodontically treated teeth (17).

7 In previous studies (18–24), teeth were decoronized, and the load was applied perpendicularly to the long axis of the root, as it was suggested that angulated loading on the crown could result in uneven stress distribution and potentially affect the accuracy of the results (21). However, a finite element analysis applying both vertical and 45° oblique loads to mandibular teeth reported no significant difference in stress distribution (29). Despite this, removing the crown may alter natural force transmission and underestimate the reinforcing effect of obturation and restorative materials. Applying a 45° load is considered more representative of clinical conditions, as it simulates the direction of masticatory and traumatic forces acting on 21 maxillary anterior teeth. Therefore, in the present study, the load was applied at a 45° angle to the palatal surface, and the crowns were left intact to preserve natural tooth structure and stress distribution, providing a more realistic simulation of clinical conditions.

TotalFill BC sealer was selected for this study because of its slight expansion upon setting, its compatibility with the single-cone obturation technique (30), and its heat-resistant nature that maintains setting time stability, allowing reliable use with the warm vertical compaction technique (31).

Previous studies (32–35) has demonstrated that calcium silicate–based sealers, particularly 1 TotalFill BC, significantly enhance the fracture resistance of endodontically treated teeth compared with epoxy resin–based sealers such as AH Plus. This improvement has been attributed to the sealer's bioactive composition, which enables chemical bonding with dentin through hydroxyapatite formation and deep penetration into dentinal tubules. In a previous study, SEM analysis confirmed this mechanism, revealing excellent adaptation of TotalFill BC to canal walls and clear penetration into dentinal tubules, whereas AH Plus exhibited interfacial gaps with no penetration (33). Moreover, Osiri et al. (35) reported that obturation with TotalFill BC combined with bioceramic-coated gutta-percha cones resulted in fracture strength values comparable to those of intact teeth. Although bioceramic-coated cones were

27 not used in the present study, the use of TotalFill BC sealer still improved the fracture resistance of the teeth, irrespective of the obturation technique employed.

9 Previous studies (18, 20, 33), in agreement with our study, reported that the highest fracture resistance values were observed in roots filled with SCT compared to WVCT regardless of the sealer type used. The SCT likely preserves dentin integrity by producing minimal stress, which may account for the higher fracture resistance observed. In addition to the vertical pressure applied during the WVCT (36), the heat generated may also influence the properties of both dentin and the sealer. It has been shown that the setting time and compressive strength of AH Plus are reduced by heat due to the loss of amine groups, which accelerates polymerization (37). However, heat has been reported not to alter the chemical structure of calcium silicate–based sealers (37). This could explain the higher fracture resistance observed in the WVCT/TotalFill BC group compared with the WVCT/AH Plus group.

Because of these characteristics of the WVCT, irreparable fractures were more frequently observed in teeth obturated using this method. The combination of vertical compaction pressure and heat application in the WVCT may generate additional stress within the root dentin. The thermal effect can also modify the mechanical properties of dentin, promoting microcrack formation and reducing structural integrity. As a result, teeth obturated with the WVCT may be more susceptible to unfavorable fracture patterns compared with those treated using the SCT.

This in vitro investigation has certain limitations. The fracture testing was performed using a continuous load of 50 N at a constant speed and direction. In clinical conditions, however, the magnitude and orientation of functional forces are variable and play a significant role in influencing tooth fracture resistance. Moreover, the periodontal ligament and surrounding tissues were not reproduced in this model, which may have affected the stress distribution pattern.

Conclusion

Within limitations, the following were concluded:

32 1. The type of sealer had a greater influence on the fracture resistance of the teeth than the obturation technique. TotalFill BC enhanced the strength of the teeth compared with AH Plus.

3

2. When clinical conditions were simulated by preserving the crown structure and applying the load at a 45° angle to the palatal surface to mimic masticatory forces on traumatized maxillary anterior teeth, the Single-Cone technique used with a bioceramic sealer demonstrated the most favorable outcomes in terms of root reinforcement and stress distribution.
3. Teeth filled with WVCT were more prone to unfavorable fracture patterns compared with those obturated using the SCT.
